# Supplementary material for: Neuromuscular blockade in mechanically ventilated pneumonia patients with moderate to severe hypoxemia: A multicenter retrospective study
Source: PLoS One. 2022 Dec 15;17(12):e0277503. doi: 10.1371/journal.pone.0277503 (PMC9754162; doi:10.1371/journal.pone.0277503)
Supplement: Attachment — Submitted filename: Response to Reviewers10.27.docx [file pone.0277503.s002.docx]

Response to Reviewer 1

Q) Reviewer #1: The use of a sedation score that is not universally used in their study is a limitation, but the data and conclusions are sound and supported by what they have observed.

A) Thank you for your thoughtful comment. Per your suggestion, we deleted the description in limitation section: “including the level of sedation (e.g., the Richmond Agitation and Sedation Scale, RASS) or delirium (e.g., the Confusion Assessment Method for the Intensive Care Unit, CAM-ICU).”

Response to Reviewer 2

Q)Reviewer #2: Thank you for addressing the comments. The authors have made appropriate adjustments to the original submission, refining and strengthening it to good effect. I have only one concern regarding excluding patients with ARDS diagnoses since it is impossible to exclude some patients with missing ARDS diagnoses remained in the included patients due to the retrospective study design. In the LUNG SAFE study, the largest international cohort among patients with ARDS, the diagnosis of ARDS was missed entirely in 40% of patients, while ARDS recognition ranged from 51% in mild ARDS to 79% in severe ARDS. In this respect, I suggest the authors keep patients with ARDS diagnosis recognition in the analysis or present both analyses, including and excluding patients with ARDS diagnosis recognition.

A) Thank you for your thoughtful commens. Per your suggestion, we reanalyzed the data including ARDS. After included of ARDS diagnosis, 90 d mortality rate was significiantly higher in NMB users (39.7% vs. 31.9%, p = 0.021). We described the revised results of the entire paper. We highlighted the main changes as yellow color. We thank you for your thoughtful comments again.
